# A cuproptosis-related lncRNA signature predicts the prognosis and immune cell status in head and neck squamous cell carcinoma

**DOI:** 10.3389/fonc.2023.1055717

**Published:** 2023-07-19

**Authors:** Xiwang Zheng, Defei Zheng, Chunming Zhang, Huina Guo, Yuliang Zhang, Xuting Xue, Zhaohui Shi, Xiangmin Zhang, Xianhai Zeng, Yongyan Wu, Wei Gao

**Affiliations:** ^1^ Shanxi Key Laboratory of Otorhinolaryngology Head and Neck Cancer, First Hospital of Shanxi Medical University, Taiyuan, Shanxi, China; ^2^ Shanxi Province Clinical Medical Research Center for Precision Medicine of Head and Neck Cancer, First Hospital of Shanxi Medical University, Taiyuan, Shanxi, China; ^3^ Department of Hematology/Oncology, Children’s Hospital of Soochow University, Suzhou, Jiangsu, China; ^4^ Department of Otolaryngology Head & Neck Surgery, First Hospital of Shanxi Medical University, Taiyuan, Shanxi, China; ^5^ Department of Otolaryngology Head & Neck Surgery, Longgang Otolaryngology Hospital, Shenzhen, Guangdong, China; ^6^ Shenzhen Institute of Otolaryngology & Key Laboratory of Otolaryngology, Longgang Otolaryngology Hospital, Shenzhen, Guangdong, China

**Keywords:** copper, cuproptosis, lncRNAs, cell death, head and neck squamous cell carcinoma, prognosis

## Abstract

**Introduction:**

The incidence of head and neck squamous cell carcinoma (HNSCC), one of the most prevalent tumors, is increasing rapidly worldwide. Cuproptosis, as a new copper-dependent cell death form, was proposed recently. However, the prognosis value and immune effects of cuproptosis-related lncRNAs (CRLs) have not yet been elucidated in HNSCC.

**Methods:**

In the current study, the expression pattern, differential profile, clinical correlation, DNA methylation, functional enrichment, univariate prognosis factor, and the immune effects of CRLs were analyzed. A four-CRL signature was constructed using the least absolute shrinkage and selection operator (LASSO) algorithm.

**Results:**

Results showed that 20 CRLs had significant effects on the stage progression of HNSCC. Sixteen CRLs were tightly correlated with the overall survival (OS) of HNSCC patients. Particularly, lnc-FGF3-4 as a single risk factor was upregulated in HNSCC tissues and negatively impacted the prognosis of HNSCC. DNA methylation probes of cg02278768 (MIR9-3HG), cg07312099 (ASAH1-AS1), and cg16867777 (TIAM1-AS1) were also correlated with the prognosis of HNSCC. The four-CRL signature that included MAP4K3-DT, lnc-TCEA3-1, MIR9-3HG, and CDKN2A-DT had a significantly negative effect on the activation of T cells follicular helper and OS probability of HNSCC. Functional analysis revealed that cell cycle, DNA replication, and p53 signal pathways were enriched.

**Discussion:**

A novel CRL-related signature has the potential of prognosis prediction in HNSCC. Targeting CRLs may be a promising therapeutic strategy for HNSCC.

## Introduction

Copper is a crucial cofactor for all organisms, and the dysregulation of copper ions can induce oxidative stress and cytotoxicity (1). A new copper-dependent cell death form was proposed and termed “cuproptosis” by Tsvetkov et al. in a recent study (2). The researchers demonstrated that copper ions can directly bind to lipoylated components in the tricarboxylic acid cycle and result in the aggregation of lipoylated protein and subsequent iron-sulfur cluster protein loss. The above events finally lead to proteotoxic stress and cell death (1, 2). Cuproptosis-related genes (CRGs) and cuproptosis-related lncRNAs (CRLs) have begun to be investigated in cancer and have exhibited promising potential in the diagnosis and prognosis prediction of cancer (3–5). However, the clinical application of CRGs in head and neck squamous cell carcinoma (HNSCC) remains unclear.

HNSCC is the sixth-most common neoplasm in the world and has 890,000 new cases reported and estimated deaths of ~450,000 in 2018. More importantly, the incidence of HNSCC is increasing rapidly and is expected to increase by 30% by 2030 (6). The molecular markers can contribute to the diagnosis and prognosis prediction in cancer clinic settings (7). Long non-coding RNAs (lncRNAs) are non-coding RNAs with longer than 200 nucleotides and lack protein-coding potential. lncRNAs play an essential role in most cellular processes and are well known as suppressed factors or oncogenes (8). Although proteins play a pivotal role in cancer diagnosis and therapy, lncRNAs also have showed the promising potential in serving as the new signature or target in early diagnosis, prognosis prediction, and treatment of many cancers (9–11). To our knowledge, there is currently no report about CRLs as signatures or targets in the diagnosis, prognosis prediction, or treatment of HNSCC.

In the current study, we primarily focused on the potentiality of CRLs as the diagnosis and prognosis prediction signature in HNSCC. A total of 501 HNSCC and 43 adjacent normal samples were analyzed in this study. The expression pattern, differential profile, clinical correlation, DNA methylation, functional enrichment, univariate prognosis factor, and immune status were analyzed. DNA methylation changes of cg02278768 (MIR9-3HG), cg07312099 (ASAH1-AS1), and cg16867777 (TIAM1-AS1) sites were correlated with HNSCC prognosis. lnc-FGF3-4 as a single risk factor was upregulated in HNSCC tissues and negatively impacted the prognosis of HNSCC. A four-CRL signature that included MAP4K3-DT, lnc-TCEA3-1, MIR9-3HG, and CDKN2A-DT was also constructed using LASSO algorithm. The four-CRL signature was found to have significantly negative effects on the immune status and prognosis of HNSC. Finally, a nomogram consisting of the four-CRL signature and clinical features was constructed for overall survival (OS) prediction of HNSCC in clinical utilization.

## Materials and methods

### Data collection

The transcriptome sequencing data of 501 HNSCC and 43 adjacent normal tissue samples were obtained from the Cancer Genome Atlas database (TCGA, https://portal.gdc.cancer.gov/). All HNSCC patients were randomly divided into the training cohort (TCGA-A) and the validation cohort (TCGA-B) in a 3:2 ratio (12). The corresponding clinical characteristics of the above patients were acquired from UCSC Xena database (https://xenabrowser.net/), and a summary of clinical characteristics is listed in **Supplemental Table S1**. Moreover, epigenomics data of HNSCC DNA methylation were also obtained from the TCGA database. Ten CRGs were manually collected from published studies (2), and the top 10 correlated lncRNAs of each CRG served as CRLs for analysis in the current study (**Supplemental Table S2**). In this study, if the information of an lncRNA was available in the NCBI (https://www.ncbi.nlm.nih.gov/gene/) or HGNC (https://www.genenames.org/) database, the official symbol was used as the name for that lncRNA. If some lncRNAs were not included into the NCBI or HGNC database but were included into the LNCipedia (https://lncipedia.org/) database, the name of these lncRNAs in the LNCipedia was used as the name for lncRNAs. If the information of a lncRNA was not available in all above three database, the ID of that lncRNA in ensembl (https://ensembl.org/) database was used as the name for lncRNA in this study.

### Cell culture of HNSCC and quantitative real-time PCR

Human embryonic kidney cells HEK293T (obtained from the China Center for Type Culture Collection, Wuhan, China) and HNSCC cell lines CAL-27, FaDu, AMC-HN-8 and WSU-HN30 (obtained from the Cell Bank of Chinese Academy of Sciences, Shanghai, China) were cultured for analysis of CRLs expression in the study. HEK293T and HNSCC cell lines were cultured in Dulbecco’s Modified Eagle’s Medium (DMEM; Life Technologies, USA) with 10% fetal bovine serum (FBS). All cell lines were maintained in a humidified atmosphere containing 5% CO2 at 37°C. 18S rRNA were used as internal controls and it’s primer sequences were sense: 5’-3’ CCTGGATACCGCAGCTAGGA and antisense:5’-3’ GCGGCGCAATACGAATGCCCC. The primer sequences of lnc-FGF3-4 were sense: 5’-3’ GTTTCCCACGCTCCCTATGA and antisense:5’-3’ TCGACAGATGAAATGAAGGCA.

### Validation of CRLs expression in laryngeal squamous cell carcinoma tissues

It were used for further validation of CRLs expression that RNA-Seq data that were consisted of 107 paired laryngeal squamous cell carcinoma (LSCC, one of the most common HNSCC) and matched adjacent normal mucosa (ANM) tissues. The samples processing, sequencing procedure and analysis methods were introduced in previous studies (13, 14).

### Analysis of expression patterns

A chord diagram of correlations among CRGs was implemented using *circlize* (v0.4.15) package in R (v4.1.0). The network of correlations amongst CRLs was plotted using the *corrr* (v0.4.3) package in R. The expression patterns of CRLs were analyzed and visualized using the hierarchical clustering method and the *pheatmap* (v1.0.12) package in R. *DESeq2* (v1.32.0) in R was employed to screen the differentially expressed CRLs (deCRLs) between HNSCC and normal tissues. The cutoff of *p* ≤ 0.05 and |log_2_Fold change| > 0 was threshold for significant deCRLs. The volcano plot of deCRLs was plotted using the *ggplot2* (v3.3.6) package in R. Student’s *t*-test was used to statistically analyze the correlations between expression levels and clinical features, and *p* ≤ 0.05 was used as the significantly differential cutoff.

### DNA methylation analysis

DNA methylation status of CRLs was analyzed using *ChAMP* (v2.22.0) package in R according to the recommended methylation chip analysis pipeline (15). The globe methylation visualization of HNSCC was implemented using *circlize* and *pheatmap*. The cutoff of *p* ≤ 0.05 and |log_2_Fold change| > 0 were used to screen the significantly differentially methylated probes (DMPs). Moreover, the effects of DMPs on survival prognosis were analyzed using univariate Cox analysis and Kaplan–Meier method. Forest plots of Cox analysis results and Kaplan–Meier curves were visualized using *forestplot* (v2.0.1) and *survival* (v3.3-1) packages in R, respectively.

### Construction of network and functional enrichment analysis

In CRL–protein-coding RNAs (pcRNAs) interaction network, the cutoff values of |*Pearson’ s r* | ≥ 0.6 and *p* ≤ 0.05 were used as the threshold of correlation. The pcRNAs were used to analyze the biological functions in HNSCC, and the enrichment analysis was performed using *clusterProfiler* (v4.0.5) package in R (16), the analysis content of which included molecular function (MF), cellular component (CC), and biological process (BP) terms of gene ontology (GO) and Kyoto encyclopedia of genes and genomes (KEGG).

### Survival analysis and construction of prediction model for prognosis

The deCRLs were analyzed using univariate Cox regression to screen the survival-related risk factors. Kaplan–Meier curves were used to validate the analysis results of Cox regression. To estimate the integrated effects of CRLs on the prognosis of HNSCC patients, LASSO algorithm was used to construct the prognostically predicted model for HNSCC patients. LASSO was implemented using the *glmnet* (v4.1-4) package in R. A formula for the risk score of CRL-related signature model was constructed, and the risk score for each patient was calculated as follows: $\text{risk score}\;=\;\sum_{i=1}^{n}\left(coe_{CRLsi}\;\times \;{exp}_{CRLsi}\right)$ , where *exp_CRLsi_* is the expression level of the risk factor *CRLsi*; *coe_CRLsi_* is the coefficient of *CRLsi*; and *n* is the quantity of CRLs in CRL-related signature. Time-dependent receiver operating characteristic (ROC) analysis was employed to estimate the ability of CRL-related signature for prognosis prediction and was performed using *timeROC* (v0.4) package in R. Furthermore, the synergistic ability of CRL-related signature model and clinical features on prognostic prediction in clinical utilization was analyzed, and a nomogram was also established. Nomogram was plotted using *rms* (v6.3-0) package in R. The calibrate curves and decision curves were used to assess the performance and reliability of the nomogram and were implemented using *rms* package and *rmda* (v1.6) package in R, respectively.

### Immune cell infiltration analysis

CIBERSORT algorithm was utilized to analyze the effects of CRL-related signature on immune cell infiltration in HNSCC. Analysis pipeline of CIBERSORT algorithm in R and leukocyte signature matrix (LM22, including 22 immune-related cells) were manually collected from published literature (17). Boxplot with jitter points of CIBERSORT analysis results was plotted using *ggpubr* (v0.4.0) package in R. *p* ≤ 0.05 was the significantly differential cutoff.

## Results

### Signification of CRLs on HNSCC progression in clinic

**Figure 1:**
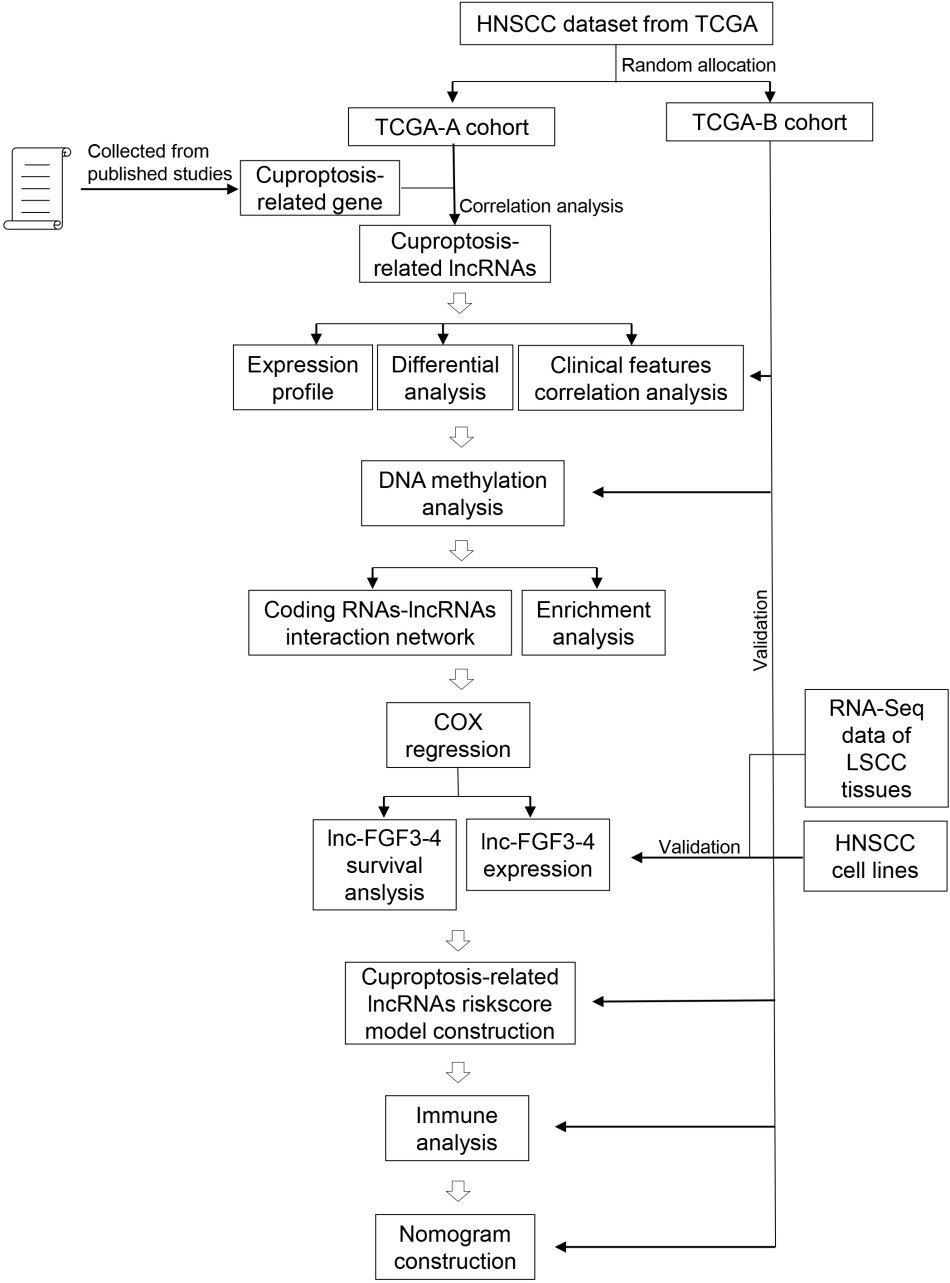
Schematic diagram of the study design.

**Figure 2:**
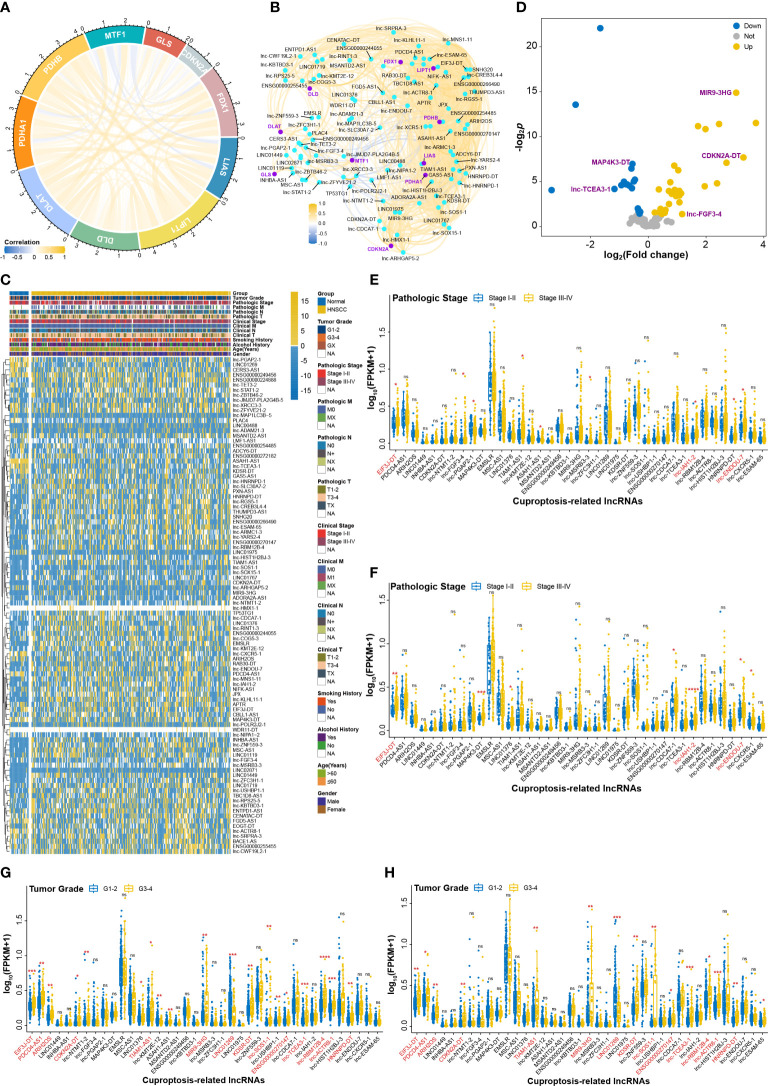
The effects of CRLs on developing and progressing HNSCC. **(A)** Correlation analysis among CRGs. **(B)** CRLs identified by correlation analysis. **(C)** The heatmap of CRLs expression between HNSCC and normal tissues. **(D)** Volcano plot of differential analysis of CRLs. **(E, F)** The effects of deCRLs on pathologic stage of HNSCC in training cohort **(E)** and in validation cohort **(F)**. **(G, H)** The effects of deCRLs on tumor grade of HNSCC in training cohort **(G)** and in validation cohort **(H)**. *p < 0.05; **p < 0.01; ***p < 0.001; ****p < 0.0001; ns, not significant.

To investigate the effects of CRLs on HNSCC, We comprehensively analyzed the underlying regulatory mechanisms of CRLs in HNSCC based on bioinformatic method and cell experiments (**Figure 1**). Ten CRGs including FDX1, LIAS, LIPT1, DLD, DLAT, PDHA1, PDHB, MTF1, GLS, and CDKN2A were manually curated for analysis in the current study (**Figure 2A**, **Supplemental Figure S1A**). The expression patterns of MTF1, GLS, and CDKN2A, especially MTF1, were opposite of those of remaining CRGs, results of which were consistent with a previous study (2). Correlation analysis was performed to identify the lncRNAs correlated with CRGs, and 99 CRLs were finally selected (**Figure 2B**, **Supplemental Figure S1B**, **Supplemental Table S2**). The heatmap in **Figure 2C** and **Supplemental Figure S1C** shows that the expression pattern between HNSCC and normal tissue was different. Differential analysis was also performed to screen the deCRLs in HNSCC tissues. There were some deCRLs in HNSCC tissues compared to normal tissues (**Figure 2D**, **Supplemental Figure S1D**). A total of 39 deCRLs respectively served as univariate factors in subsequent clinical correlation analysis. **Figures 2E, F** show that the expressions of EIF3J-DT, lnc-IAH1-2, and lnc-ENDOU-7 were upregulated in the advanced pathologic stage (stage III–IV) in HNSCC patients. EIF3J-DT, PDCD4-AS1, ARIH2OS, CDKN2A-DT, TIAM1-AS1, MIR9-3HG, LINC01269, KDSR-DT, lnc-SOS1-1, ENSG00000270147, lnc-TCEA3-1, lnc-RBM12B-4, lnc-ACTR8-1, and HNRNPD-DT had a tight correlation with the development of tumor grade in HNSCC (**Figures 2G, H**). lnc-KMT2E-12 had a higher expression level in clinical stage III–IV than that in clinical stage I–II (**Supplemental Figures S1E, F**). Moreover, EMSLR, lnc-KMT2E-12 and lnc-SOS1-1, CDKN2A-DT and lnc-SOS1-1, MIR9-3HG, EIF3J-DT, TIAM1-AS1, MIR9-3HG, lnc-USHBP1-1, ENSG00000270147, lnc-CDCA7-1, lnc-TCEA3-1, and HNRNPD-DT had a close correlation with clinical N stage, clinical T stage, age, and gender, respectively (**Supplemental Figures S2A–H**). In summary, correlation analysis between clinical features and CRLs revealed that CRLs have a potential influence on the initiation and progression of HNSCC in clinical application.

### DNA methylation status of CRLs in HNSCC

**Figure 3:**
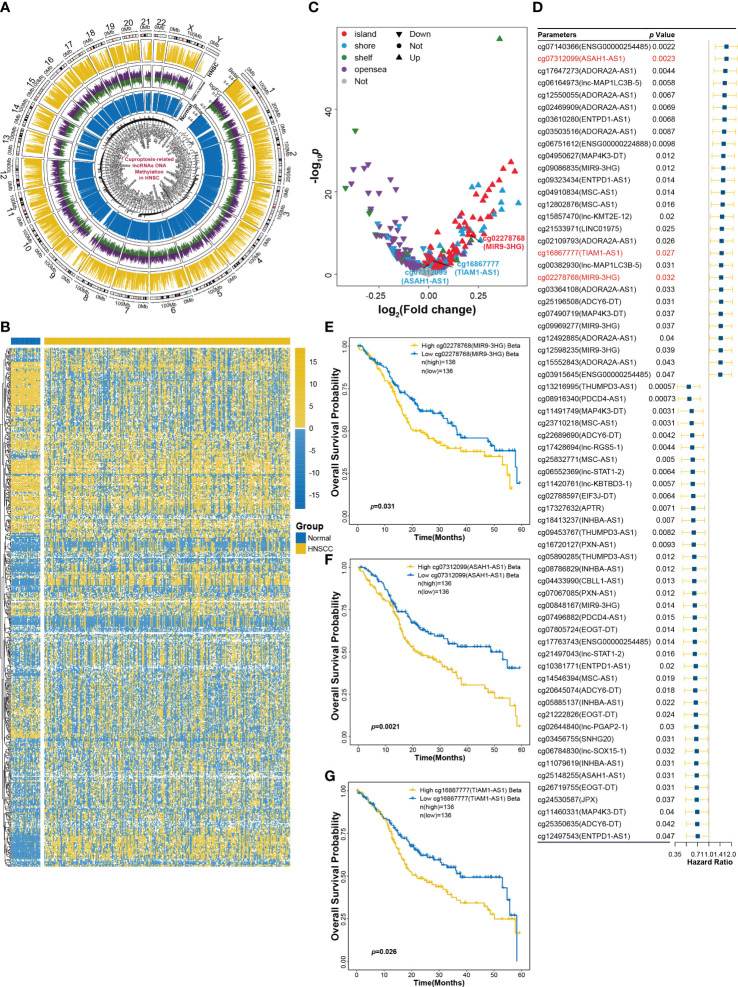
DNA methylation analysis of CRLs in HNSCC. **(A)** Methylation changes in all chromosomes of HNSCC tissues. **(B)** The heatmap of CRLs DNA methylation levels between HNSCC and normal tissues. **(C)** Volcano plot of differential analysis of CRLs DNA methylation. **(D)** Univariant Cox regression analysis for CRLs DNA methylation as an independent prognostic factor. **(E–G)** Kaplan−Meier OS curves for patients with HNSCC based on cg02278768 (MIR9-3HG) **(E)**, cg07312099 (ASAH1-AS1) **(F)** and cg16867777 (TIAM1-AS1) **(G)** in training cohort.

DNA methylation plays an essential role in gene expression and tissue differentiation and can act as the signature for the evaluation of immune response and prognosis of cancers (18). To assess DNA methylation status of CRLs in HNSCC tissue, K450 Illumina DNA methylation arrays data of HNSCC obtained from TCGA database were analyzed. **Figure 3A** shows that DNA methylation changes were widespread on chromosomes of HNSCC tissues. Furthermore, DNA methylation arrays showed that DNA region of CRLs had different methylated levels between HNSCC and normal tissues (**Figure 3B**). Differential analysis was also performed and indicated some methylated sites that showed significantly methylated changes in CRLs DNA region (**Figure 3C**). In addition, effects of CRL DNA methylation on survival prognosis of HNSCC patients were analyzed using univariate Cox regression method. Results showed that 66 methylated sites correlated with the prognosis of HNSCC patients (**Figure 3D**). The OS probability of HNSCC patients in 5-year survival was also analyzed to validate the results of univariate Cox regression. Kaplan–Meier curves showed that the methylation of cg02278768 (MIR9-3HG), cg07312099 (ASAH1-AS1), and cg16867777 (TIAM1-AS1) sites had significant effects on survival probability in the training cohort (**Figures 3E–G**) and the validation cohort (**Supplemental Figures S3A–C**).

### Construction of CRL–pcRNAs interaction network and enrichment analysis

**Figure 4:**
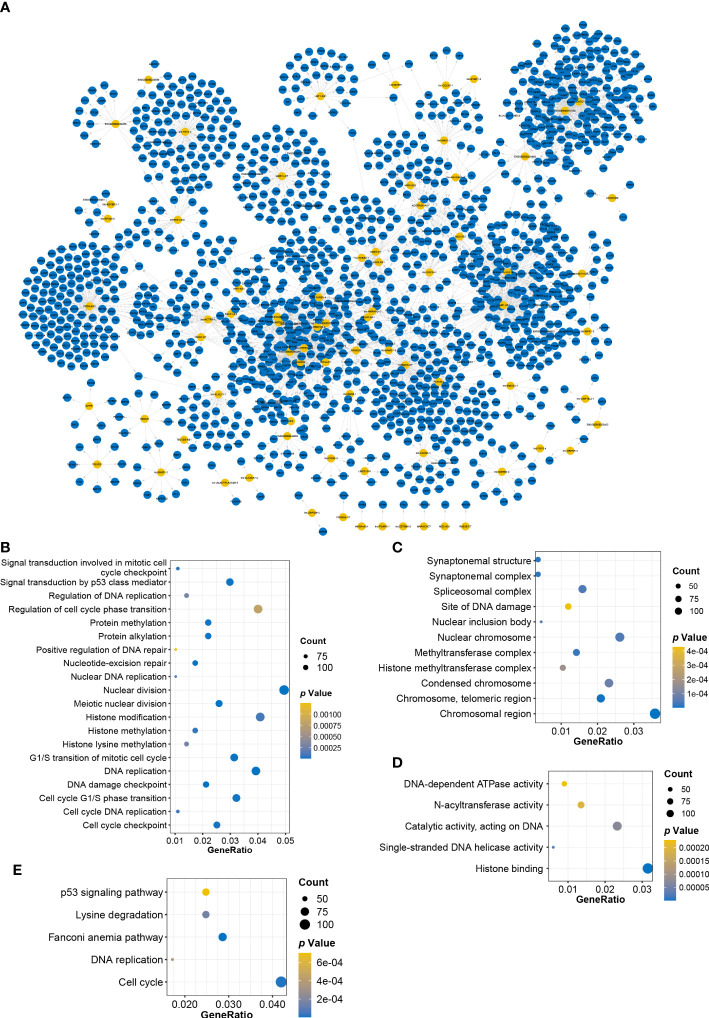
Constructing CRLs−pcRNAs interaction network and enrichment analysis. **(A)** Constructing CRLs−pcRNAs interaction network. Yellow: CRLs, blue: pcRNAs. **(B–D)** BP **(B)**, CC **(C)** and MF **(D)** terms of GO enrichment analysis. **(E)** The dotplot of KEGG enrichment analysis.

The biological functions of lncRNAs can be fulfilled through the regulation of targeted genes (19). To investigate the functional pathway of CRLs, correlation analysis was performed to screen CRL-related pcRNAs and construct the CRL–pcRNA interaction network. Based on the cutoff of |*Pearson’ s r*| ≥ 0.6 and *p* ≤ 0.05, 1475 CRL-related pcRNAs were selected (**Supplemental Table S3**), and a CRL–pcRNA interaction network was also finally established (**Figure 4A**). CRL-related pcRNAs were subsequently analyzed for functional enrichment. Results of GO enrichment analysis, including MF, CC, and BP terms, showed that pcRNAs regulated by CRLs mainly enriched in cell cycle, DNA replication, histone modification, nuclear division, and p53 signal pathway (**Figures 4B–D**). Furthermore, KEGG analysis validated the results that pcRNAs mainly enriched in cell cycle, DNA replication, and p53 signal pathway obtained from GO analysis (**Figure 4E**).

### lnc-FGF3-4 was upregulated and affected survival prognosis of HNSCC patients

**Figure 5:**
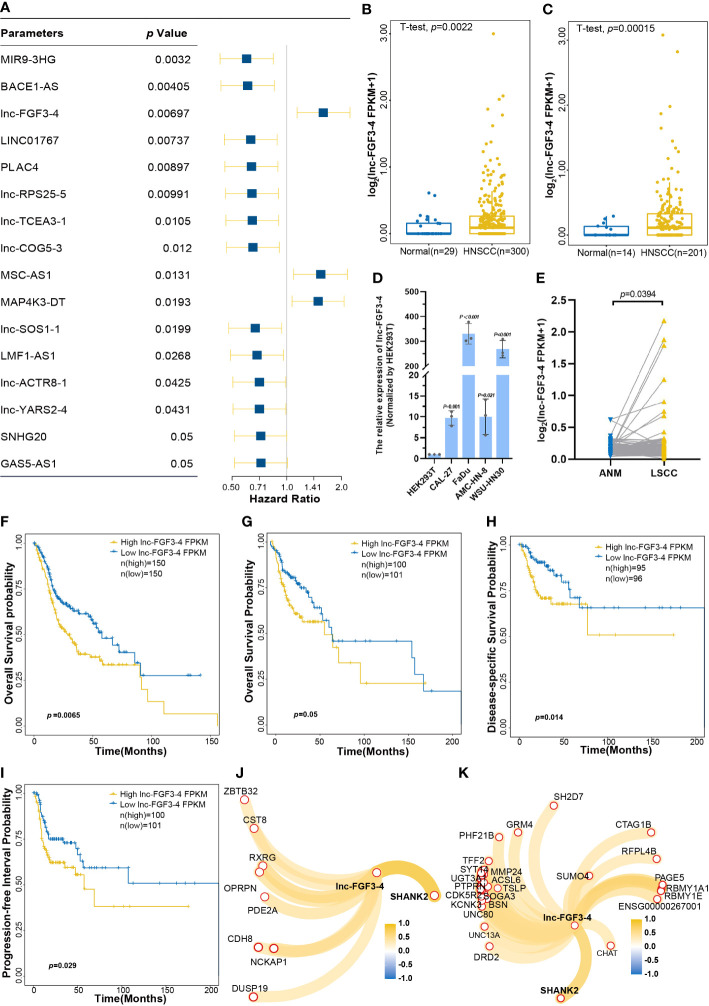
The effects of lnc-FGF3-4 on prognosis of HNSCC. **(A)** Univariant Cox regression analysis for CRLs as the independent prognosis risk factor. **(B, C)** Boxplot of lnc-FGF3-4 expression levels between HNSCC and normal tissues in training cohort **(B)** and in validation cohort **(C)**. **(D)** The expression leves of lnc-FGF3-4 in HNSCC cell lines. **(E)** The expression leves of lnc-FGF3-4 in 107 paired LSCC and matched ANM tissues. **(F)** Kaplan−Meier curves for OS of HNSCC based on lnc-FGF3-4 expression in TGCA-A cohort. **(G–I)** Kaplan-Meier curves of OS **(G)**, DSS **(H)** and PFI **(I)** of HNSCC based on lnc-FGF3-4 expression in TGCA-B cohort. **(J, K)** Protein-coding RNAs correlated with lnc-FGF3-4 expression in TGCA-A **(J)** and TGCA-B **(K)** cohort, |*Pearson’ s r*≥ 0.5 and *p* ≤ 0.05.

To evaluate the effects of each deCRL on HNSCC prognosis, univariate Cox regression was performed to analyze the correlation between survival prognosis and continuous changes of deCRL expression. Cox analysis showed that a total of 16 CRLs that included MIR9-3HG, BACE1-AS, lnc-FGF3-4, LINC01767, APLC4, lnc-RPS25-5, lnc-TCEA3-1, lnc-COG5-3, MSC-AS1, MAP4K3-DT, lnc-SOS1-1, LMF1-AS1, lnc-ACTR8-1, lnc-YARS2-4, SNHG20, and GAS5-AS1 were correlated with HNSCC prognosis (**Figure 5A**). Especially, lnc-FGF3-4 as a high-risk factor for HNSCC was upregulated in HNSCC tissues (**Figures 5B, C**). It also were confirmed that the upregulated expression of lnc-FGF3-4 in HNSCC cell lines, which included CAL-27, FaDu, AMC-HN-8 and WSU-HN30 cells (**Figure 5D**). Furthermore, the RNA-Seq data of 107 paired LSCC and matched ANM tissues showed the high expression of lnc-FGF3-4 in LSCC tissues (**Figure 5E**). Subsequently, Kaplan–Meier curves were used to validate the analysis results of Cox regression. **Figure 5F** shows that OS probability of HNSCC patients in high-lnc-FGF3-4-expression group was lower than those in low-lnc-FGF3-4-expression group. The upregulation of lnc-FGF3-4 was also observed to have a negative effect on OS probability of HNSCC patients in TCGA-B cohort (**Figure 5G**). The elevated expression of lnc-FGF3-4 also exhibited the adverse impacts on disease-specific survival (DSS) and progression-free interval (PFI) of HNSCC patients in the validation cohort (**Figures 5H, I**). Furthermore, lnc-FGF3-4 showed the significant correlations with SHANK2 in expression levels in both TCGA-A and TCGA-B cohort (**Figures 5J, K**).

### Construction and validation of CRL-related signature for OS prediction of HNSCC patients

**Figure 6:**
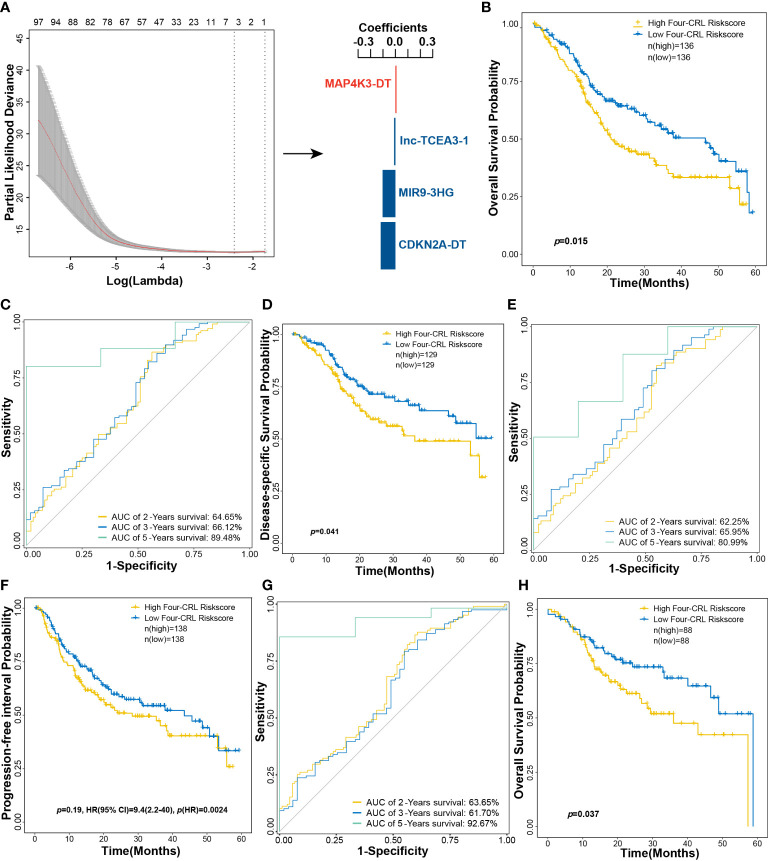
A four-CRL signature was constructed based on LASSO method. **(A)** constructing multi-CRL signature for OS of HNSCC. **(B)** Kaplan−Meier OS curves for HNSCC patients based on the four-CRL signature in training cohort. **(C)** ROC curves of 2-, 3-, 5-year OS survival according to the four-CRL signature. **(D)** Kaplan−Meier DSS curves for HNSCC patients based on the four-CRLs signature in training cohort. **(E)** ROC curves of 2-, 3-, 5-year DSS survival according to the four-CRL signature. **(F)** Kaplan−Meier PFI curves for HNSCC patients based on the four-CRL signature in training cohort. **(G)** ROC curves of 2-, 3-, 5-year PFI survival according to the four-CRL signature. **(H)** Kaplan-Meier OS curves for HNSCC patients based on the four-CRL signature in validation cohort.

The performance of multi-gene model for prognosis prediction is better than the single-gene model (20). To evaluate the integrated effects of CRLs on OS probability of HNSCC, CRLs were used to establish a predicted model based on the LASSO algorithm. A four-CRL signature, with MAP4K3-DT, lnc-TCEA3-1, MIR9-3HG, and CDKN2A-DT, was finally constructed, and the risk score of HNSCC patients was expressed as follows: risk scores = (0.0079 × *exp_MAP4K3-DT_*) + (−0.0108 × *exp_lnc-TCEA3-1_*) + (−0.1030 × *exp_MIR9-3HG_*) + (−0.1181 × *exp_CDKN2A-DT_*) (**Figure 6A**). High-risk-score group of the four-CRL signature exhibited a significantly negative effect on OS probability of HNSCC patients in the 5-year survival (**Figure 6B**). ROC curves were used to assess the sensitivity and specificity of the four-CRL signature in different lengths of survival prognosis. The area under the curve (AUC) of ROC curve was 64.65%, 66.12%, and 89.48% in 2-, 3-, and 5-year OS prediction, respectively (**Figure 6C**). The four-CRL signature also had a poor impact on the probability of DSS, and the AUC of ROC for DSS prediction was 62.25%, 65.95%, and 80.99% in 2-, 3-, and 5-year survival, respectively (**Figures 6D, E**). Although the effect of the four-CRL signature on PFI was not statistically significant in Kaplan–Meier estimate, it was highly significant that the four-CRL signature as a risk factor had an adverse impact on PFI probability in univariate analysis (*p*(HR) = 0.0024, **Figure 6F**). In addition, the AUC of ROC for PFI prediction was 63.65%, 61.70%, and 92.67% in 2-, 3-, and 5-year survival, respectively (**Figure 6G**). Finally, the adverse effect of the four-CRL signature on OS probability was validated in the validation cohort (**Figure 6H**).

### High-risk scores of the four-CRL signature suppressed the immune status of HNSCC

**Figure 7:**
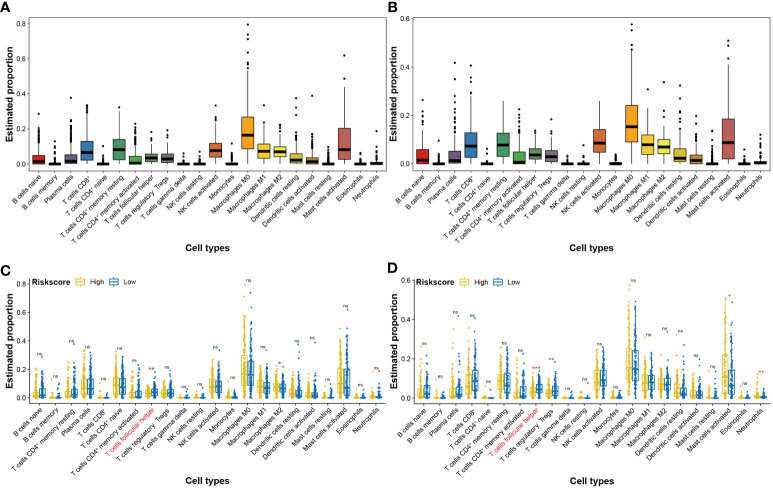
Effects of the four-CRL signature on immune cells status in HNSCC tissues. **(A, B)** The proportion of 22 immune cells in HNSCC tissues in training cohort **(A)** and in validation cohort **(B)**. **(C, D)** The proportional difference of 22 immune cells between high and low four-CRL signature risk-score in training cohort **(C)** and in validation cohort **(D)**. *p < 0.05; **p < 0.01; ***p < 0.001; ns, not significant.

In the initiation and development of cancer, the peripheral immune system is often weak (21). To assess the effects of the four-CRL signature on the immune status of HNSCC, CIBERSORT method was used to analyze the correlation between the four-CRL signature and immune cell abundance. **Figure 7A** displays the different proportions of 22 immune-related cells in HNSCC tissues. The distributed trends of 22 immune-related cells in TCGA-B cohort have confirmed the analysis results in the training cohort (**Figure 7B**). T cells follicular helper is a specific subgroup of CD4^+^ T cells that help B cells produce antibodies against foreign antigens (22). The proportion of T cells follicular helper was significantly suppressed in high-risk score of the four-CRL signature (**Figure 7C**). In addition, trends of T cells follicular helper weakened by the four-CRL signature were confirmed in the validation cohort (**Figure 7D**).

### Construction of a CRL-related nomogram for clinical application

**Figure 8:**
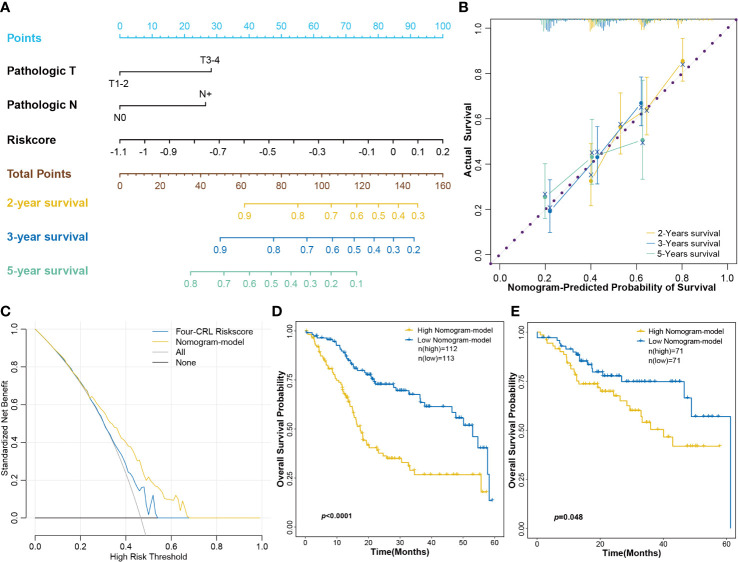
Constructing a nomogram consisted of the four-CRL signature and clinical characters. **(A)** The nomogram for OS survival prediction of HNSCC patients. **(B)** The calibrate curves evaluated the prediction ability of nomogram model. **(C)** The decision curves for evaluating the four-CRL signature and nomogram model in OS prediction. **(D, E)** Kaplan−Meier curves for OS based on the nomogram model in training cohort **(D)** and in validation cohort **(E)**.

Combining biomarkers and clinical features can improve the efficiency of disease diagnosis or prognosis prediction compared with that obtained using a single indicator (23). To screen the clinical features correlated with HNSCC prognosis, univariate Cox regression was employed for risk factor analysis. Results revealed that pathologic T and pathologic N stages were significantly correlated with the prognosis of HNSCC (**Supplemental Figure S4**). In this study, a nomogram consisting of the four-CRL signature and pathologic T and pathologic N stages was constructed for survival prognosis prediction in clinical use (**Figure 8A**). The calibrate curves were used to evaluate the prediction ability of the nomogram model, and the nomogram exhibited a great prognostic predictive validity of OS in 2-, 3-, and 5-year survival (**Figure 8B**). Moreover, decision curves showed the net benefit of the four-CRL signature and nomogram model in OS prediction (**Figure 8C**). **Figure 8D** shows the high-risk group in the nomogram model had significantly negative effects on OS probability in 5-year survival. TCGA-B cohort validated the performance of the nomogram model in OS prediction in the training cohort (**Figure 8E**).

## Discussion

In this study, 99 CRLs were analyzed in 501 HNSCC and 43 normal tissue samples. The expression pattern, differential profile, clinical correlation, DNA methylation, functional enrichment, univariate prognosis factor, and the immune status were analyzed. A four-CRL signature for OS probability prediction was constructed using LASSO algorithm. Furthermore, a nomogram consisting of the four-CRL signature and clinical features was established for clinical utilization of HNSCC.

lncRNAs play a crucial role in the regulation of most cellular processes at various levels, such as epigenetic modification, and the regulation of transcription, post-transcription, translation, and post-translation. lncRNAs also regulate the initiation and progression in cancers of diverse tissues through various molecular mechanisms (9). Certain deCRLs, namely, EIF3J-DT, lnc-IAH1-2, lnc-ENDOU-7, PDCD4-AS1, ARIH2OS, CDKN2A-DT, TIAM1-AS1, MIR9-3HG, LINC01269, KDSR-DT, lnc-SOS1-1, ENSG00000270147, lnc-TCEA3-1, lnc-RBM12B-4, lnc-ACTR8-1, HNRNPD-DT, lnc-KMT2E-12, EMSLR, lnc-USHBP1-1, and lnc-CDCA7-1 were significantly correlated with clinical features in the current study. In the lncRNAs above, EIF3J-DT, PDCD4-AS1, CDKN2A-DT, MIR9-3HG, LINC01269, and EMSLR have been reported to play a critical role in the regulation of cancers (24–29). In particular, a previous study revealed that LINC01269 could be a tumor inhibitor as it triggered therapeutic efficacy in HNSCC (28), which validated the analysis result that LINC01269 was significantly correlated with the progression of HNSCC tumor grade in the current study. These results fully demonstrated that CRLs have potential effects on the initiation and progression of HNSCC in clinical applications and can be used to construct the progression or prognosis prediction model.

DNA methylation, as one of the epigenetic modifications, regulates gene expression without changing the DNA sequence and is actively involved in the progression of cancers (30). DNA methylation has also been reported to impact the stage progression of HNSCC (31). In the current study, many methylation probes that targeted DNA sequence of CRLs brought significant changes in HNSCC tissues. More particularly, it was identified that DNA methylation changes of cg02278768 (MIR9-3HG), cg07312099 (ASAH1-AS1), and cg16867777 (TIAM1-AS1) sites were correlated with the prognosis of HNSCC. Based on the published literature, the expression of MIR9-3HG was regulated by its DNA methylation changes in prostate cancer (32). MIR9-3HG was hypomethylated in DNA sequence and upregulated in the transcriptome level in this study. The results of DNA methylation analysis revealed that DNA methylation levels of CRLs might regulate their expression levels. Particularly, DNA hypomethylation elevated the expression of MIR9-3HG, and the change of methylated site in MIR9-3HG DNA sequence even affected the OS of HNSCC patients.

Certain CRLs have already been used to analyze the effects on the progression of cancers and predict the prognosis of patients (5, 33, 34). However, the regulatory mechanisms of CRLs in HNSCC are still unclear until now. In our study, 99 CRLs were utilized for functional enrichment analysis. Results of GO and KEGG analysis showed that CRLs mainly enriched in histone modification, nuclear division, cell cycle, DNA replication, and p53 signal pathway. The dysregulation of histone modification can generate the inappropriate activation of oncogenes or tumor suppressors (35). Abnormal nuclear division is an important event in the development of cancers and can be used to assess the initiation of cancers (36, 37). DNA replication stress is one of the hallmarks of cancers, induced by tumor driver genes to result in a rapid proliferation of tumor cells (38). It is well known that disturbed regulation of the cell cycle is one of the main causes of tumorigenesis (39). P53 signal pathway has preeminent importance in regulating cell proliferation, and its mutation directly promotes the initiation of cancers (40). These results suggest that CRLs play an essential role in the regulation of cancers and have the potential as biomarkers or targets in the prediction and therapy of cancers.

In our study, independent risk factors were identified using univariate Cox proportional hazards models. A total of 16 CRLs, which included MIR9-3HG, BACE1-AS, lnc-FGF3-4, LINC01767, APLC4, lnc-RPS25-5, lnc-TCEA3-1, lnc-COG5-3, MSC-AS1, MAP4K3-DT, lnc-SOS1-1, LMF1-AS1, lnc-ACTR8-1, lnc-YARS2-4, SNHG20, and GAS5-AS1 had significant effects on OS probability of HNSCC patients. The overexpression of BACE1-AS promoted the invasive and metastatic capacity of hepatocellular carcinoma (41). MSC-AS1 enhances the proliferation and glycolysis of gastric cancer (42). MAP4K3-DT was positively correlated with the VEGF-C/VEGFR3-induced lymph node metastasis of bladder cancer (43). SNHG20 is an aberrant expression in various cancers and promotes the development and progression of tumors, such as hepatocellular carcinoma, ovarian cancer, colorectal cancer, and bladder cancer (44). Downregulation of GAS5-AS1 can suppress the development and metastasis of cervical cancer (45). Particularly, a novel lncRNA lnc-FGF3-4 as a single risk factor was upregulated in HNSCC tissues and had a significantly negative impact on the patient’s prognosis. Deletion of GLS confer sensitivity to copper-induced cell death in cuproptosis mechanism (2). Because lnc-FGF3-4 is a significantly expression-associated lncRNA with GLS, lnc-FGF3-4 may maintain the cells survival by elevating the expression of GKL to suppress the copper-induced cell death. Moreover, lnc-FGF3-4 also show a significant positive correlation with SHANK2 in expression levels. SHANK2 is upregulation in cancer cells and can suppress the Hippo signaling pathway (46). lnc-FGF3-4 may promote tumor cell proliferation by upregulating SHANK2 to inhibit the Hippo signaling pathway. The above results reveal that CRLs could affect the OS probability of HNSCC patients and exhibit promising potential in the prognosis prediction of HNSCC.

The multi-gene model for prognosis prediction performs better than the single-gene model (20). We have constructed a four-CRL signature that included MAP4K3-DT, lnc-TCEA3-1, MIR9-3HG, and CDKN2A-DT using the LASSO algorithm. In the 2-, 3-, and 5-year survival prediction, the AUC values of the four-CRL risk score for OS were 64.65%, 66.12%, and 89.48%, for DSS were 62.25%, 65.95%, and 80.99%, and for PFI were 63.65%, 61.70%, and 92.67%, respectively. MAP4K3-DT, MIR9-3HG, and CDKN2A-DT have been reported to have the predictive ability of cancer prognosis and regulate the development and progression of cancers (26, 27, 43). The knockout of FDX1 resulted in cancer cells becoming sensitive to the copper ion concentration, conversely, the knockout of CDKN2A improved the tolerance of cancer cells to copper ion (2). As the expression-correlated lncRNAs of FDX1, MAP4K3-DT is significantly downregulated in HNSCC, and perhaps suppresses the death of HNSCC cells through attenuating the expression of FDX1 that a core gene in cuproptosis. Moreover, MIR9-3HG and CDKN2A-DT, the expression-correlated lncRNAs of CDKN2A, are upregulated in HNSCC and may be improve the resistance of HNSCC cells to copper-induced cell death through regulating expression of CDKN2A.

Immunoevasion and immunosuppression are ubiquitous in malignant cancer and serve as emerging hallmarks (40). HNSCC has an immunosuppressive tumor microenvironment that is accompanied by low tumor‐infiltrating lymphocytes (47). In our study, the proportion of T cells follicular helper was significantly suppressed in high-risk score of the four-CRL signature. T cells follicular helper is a specific subgroup of CD4^+^ T cells that helps B cells produce antibodies against foreign antigens (22). Some recent studies indicated that the mediatory effect of follicular helper T cells on diverse cancers. For instance, a functional analysis in non-small cell lung carcinoma revealed that follicular helper T cells were capable of promoting the differentiation of regulatory B cells and CD14^+^ human leukocyte antigen (HLA) - DR - cells. Furthermore, a analysis in the same study uncovered a negative association between follicular helper T cells and disease-free survival in patients with non-small cell lung carcinoma (48). One study uncovered that the infiltration of follicular helper T cells in HPV^+^ HNSCC patients was higher than who in HPV^-^ HNSCC patients cohort (49). Moreover, a investigation based on HNSCC patients showed genes expression signature related with CD4^+^T follicular helper cells could affect the progression-free survival of HNSCC patients (50). These results suggest that follicular helper T cells may play an important role in the initiation and development of HNSCC. Therefore, the effect of the four-CRL on follicular helper T cells may be one of its regulatory pathways in HNSCC. Combining molecular biomarkers and clinical characteristics can improve the efficiency of disease diagnosis or prognosis prediction compared with that obtained using a single indicator (23). We also established a nomogram consisting of a four-CRL signature and clinical characteristics for clinical application. The calibrate curves and decision curves have validated the prediction ability of the nomogram model in HNSCC. The above results fully demonstrate that the four-CRL signature has promising potential in the prognosis prediction of HNSCC and as the treatment targets in clinical therapy.

Notwithstanding its novel findings, the current study has a few limitations. First, the cohorts of HNSCC patients analyzed in our study were retrospective data based on the public database. Our prospective multi-center clinical data will be used to further verified the prognostically predictive model in the future. Second, it is inevitable that relying on only a limited number of genes to build a prognostic model has the inherent weaknesses. As the public data grows, more CRLs will also be incorporated in future studies. Finally, it must be emphasized here that the correlation between the four-CRL signature and promotion of HNSCC has not yet been experimentally addressed. The experimental investigation of CRLs in HNSCC will act as a new object for further exploration.

## Conclusion

In this study, the expression pattern, differential profile, clinical correlation, DNA methylation, functional enrichment, univariate prognosis factor, and immune status were analyzed. DNA methylation changes of cg02278768 (MIR9-3HG), cg07312099 (ASAH1-AS1), and cg16867777 (TIAM1-AS1) sites were correlated with the prognosis of HNSCC. lnc-FGF3-4, as a single risk factor, was upregulated in HNSCC tissues and negatively impacted the prognosis of HNSCC. A four-CRL signature that included MAP4K3-DT, lnc-TCEA3-1, MIR9-3HG, and CDKN2A-DT was constructed. This four-CRL signature has exhibited significantly negative effects on the immune status and survival prognosis of HNSCC. Finally, a nomogram consisting of the four-CRL signature and clinical features was also established for clinical applications. CRLs have exhibited promising potential in the prognosis prediction of HNSCC and as the treatment targets in immunotherapy.

## Data availability statement

Publicly available datasets were analyzed in this study. TCGA data can be found here: https://portal.gdc.cancer.gov/, https://xenabrowser.net/. RNA-seq data of 107 paired LSCC can be found at GEO (https://www.ncbi.nlm.nih.gov/geo/) with accession number GSE127165 and GSE130605.

## Author contributions

XWZ obtained data, calculated data, plotted figure and drafted the manuscript. DZ and CZ performed the statistical analysis, drafted and revised the manuscript. HG, YZ, XX, ZS, and XMZ calculated data, cultured cells, quantitated RNAs, and plotted figures. XHZ, YW, and WG designed the study, administrated the project, and revised the manuscript. All authors reviewed and approved the final manuscript. All authors contributed to the article and approved the submitted version.

## Supplementary material

Supplementary Figure 1 The validation of CRLs’s role in developing and progressing HNSCC. **(A)** Correlation analysis of among CRGs in validation cohort. **(B)** Correlation analysis among CRLs in validation cohort. **(C)** The heatmap of CRLs expression between HNSCC and normal tissues in validation cohort. **(D)** Volcano plot of differential analysis of CRLs in validation cohort. **(E, F)** The effects of deCRLs on clinical stage of HNSCC in training cohort **(E)** and in validation cohort **(F)**. **p* < 0.05; ***p* < 0.01; ****p* < 0.001; *****p* < 0.0001; ns, not significant.

Supplementary Figure 2 The effects of CRLs on clinical features of HNSCC patients. **(A, B)** The effects of deCRLs on clinical T stage of HNSCC in training cohort **(A)** and validation cohort **(B)**. **(C, D)** The effects of deCRLs on clinical N stage of HNSCC in training cohort **(C)** and validation cohort **(D)**. **(E, F)** The correlation between deCRLs and age of HNSCC patients in training cohort **(E)** and validation cohort **(F)**. **(G, H)** The correlation between deCRLs and gender of HNSCC patients in training cohort **(G)** and validation cohort **(H)**. **p* < 0.05; ***p* < 0.01; ****p* < 0.001; *****p* < 0.0001; ns, not significant.

Supplementary Figure 3 DNA methylation analysis validation of CRLs in HNSCC in validation cohort. **(A–C)** Kaplan−Meier OS curves for HNSCC patients based on cg02278768 (MIR9-3HG) **(A)**, cg07312099 (ASAH1-AS1) **(B)** and cg16867777 (TIAM1-AS1) **(C)** methylation levels in validation cohort.

Supplementary Figure 4 Univariant Cox regression analysis of OS based on clinical features.

Supplementary Table 1 Clinical characters of HNSCC patients in the current study.

Supplementary Table 2 A list of cuproptosis-related lncRNAs.

Supplementary Table 3 A list of protein conding RNAs correlated with CRLs.
