# Supplementary figures and images for: A cuproptosis-related lncRNA signature predicts the prognosis and immune cell status in head and neck squamous cell carcinoma
Source: Front Oncol. 2023 Jul 19;13:1055717. doi: 10.3389/fonc.2023.1055717 (PMC10394648; doi:10.3389/fonc.2023.1055717)

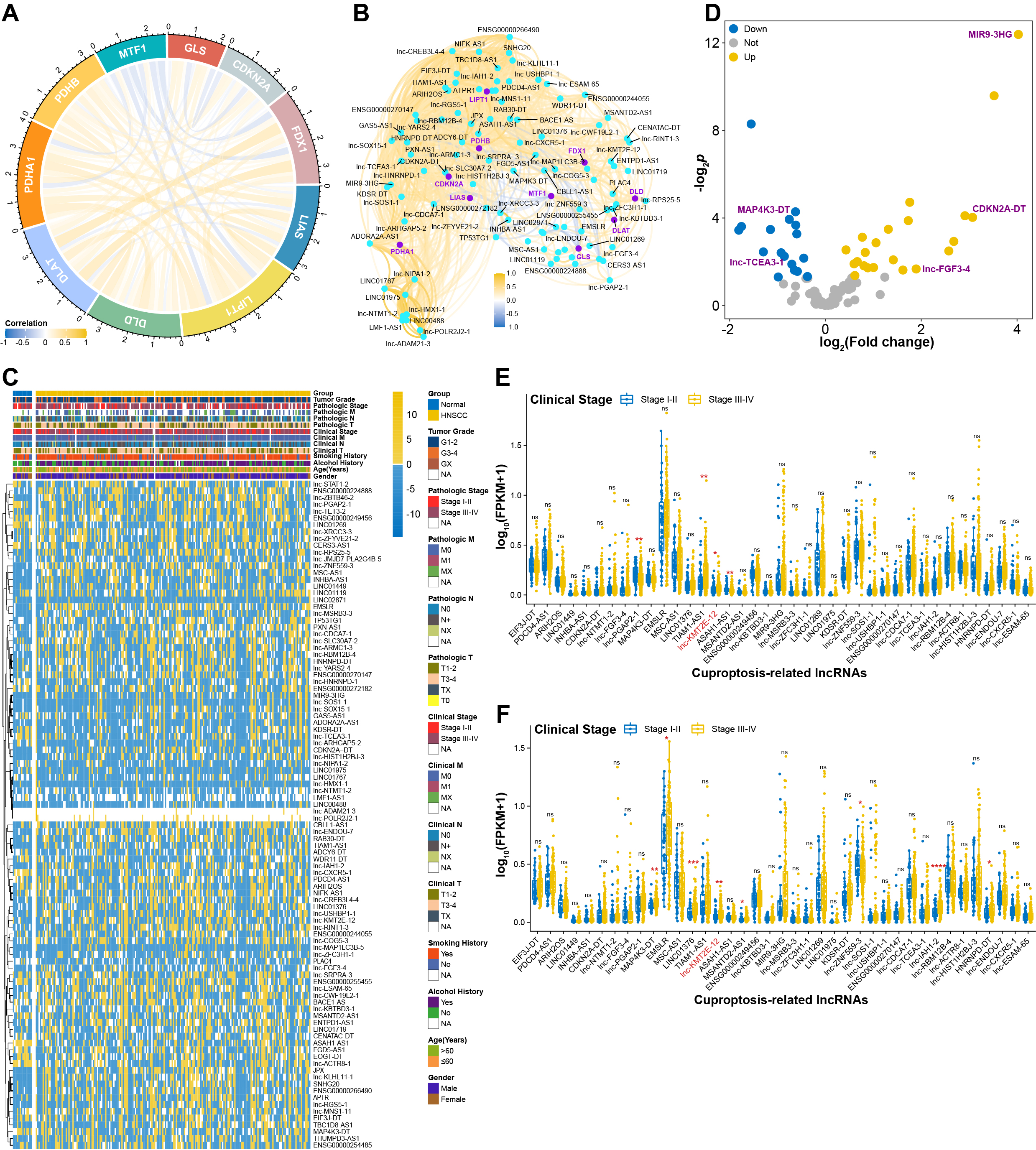

Supplement: Supplementary Figure 1 — The validation of CRLs’s role in developing and progressing HNSCC. (A) Correlation analysis of among CRGs in validation cohort. (B) Correlation analysis among CRLs in validation cohort. (C) The heatmap of CRLs expression between HNSCC and normal tissues in validation cohort. (D) Volcano plot of differential analysis of CRLs in validation cohort. (E, F) The effects of deCRLs on clinical stage of HNSCC in training cohort (E) and in validation cohort (F). *p < 0.05; **p < 0.01; ***p < 0.001; ****p < 0.0001; ns, not significant. [file Image_1.jpeg]

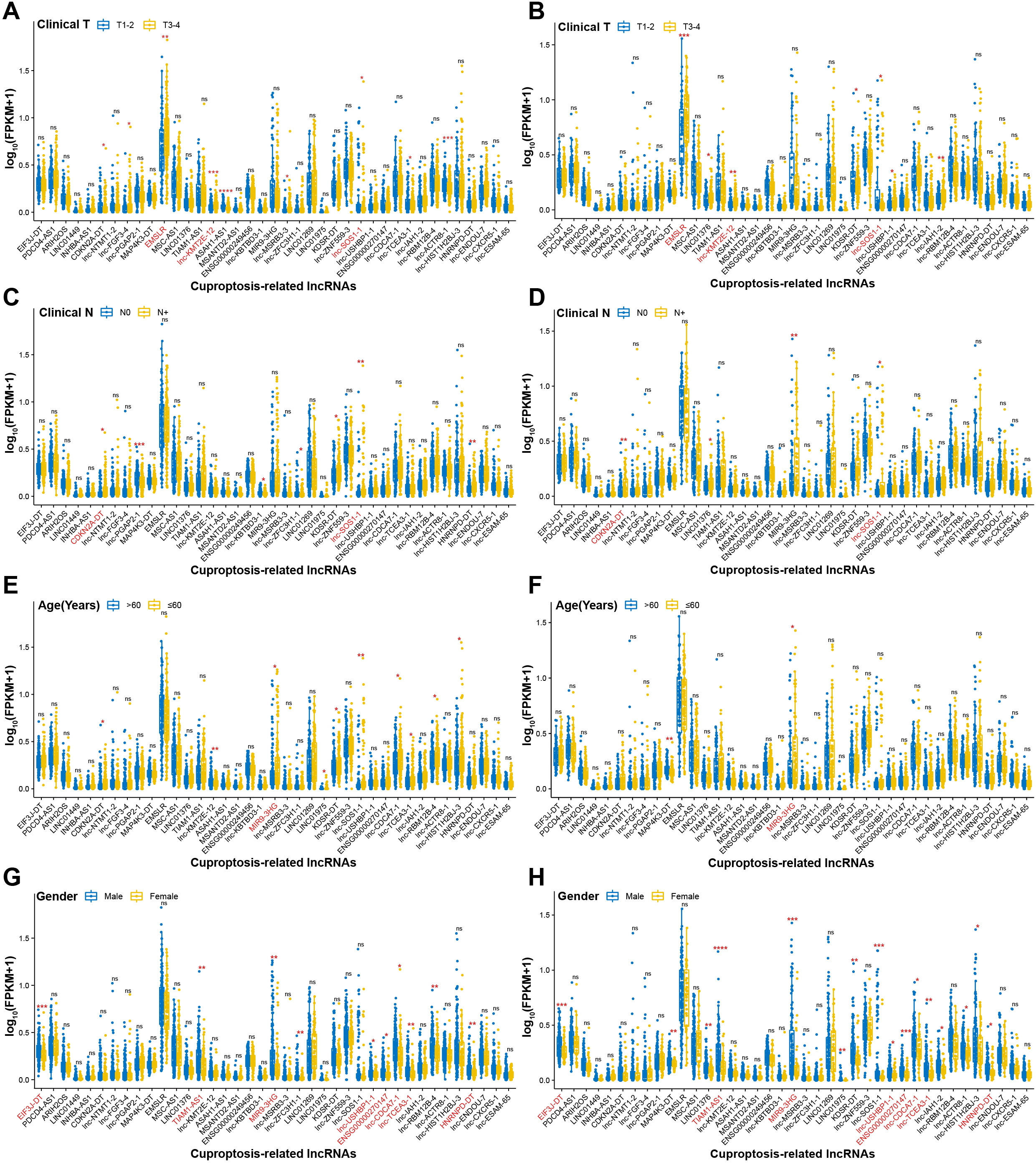

Supplement: Supplementary Figure 2 — The effects of CRLs on clinical features of HNSCC patients. (A, B) The effects of deCRLs on clinical T stage of HNSCC in training cohort (A) and validation cohort (B). (C, D) The effects of deCRLs on clinical N stage of HNSCC in training cohort (C) and validation cohort (D). (E, F) The correlation between deCRLs and age of HNSCC patients in training cohort (E) and validation cohort (F). (G, H) The correlation between deCRLs and gender of HNSCC patients in training cohort (G) and validation cohort (H). *p < 0.05; **p < 0.01; ***p < 0.001; ****p < 0.0001; ns, not significant. [file Image_2.jpeg]

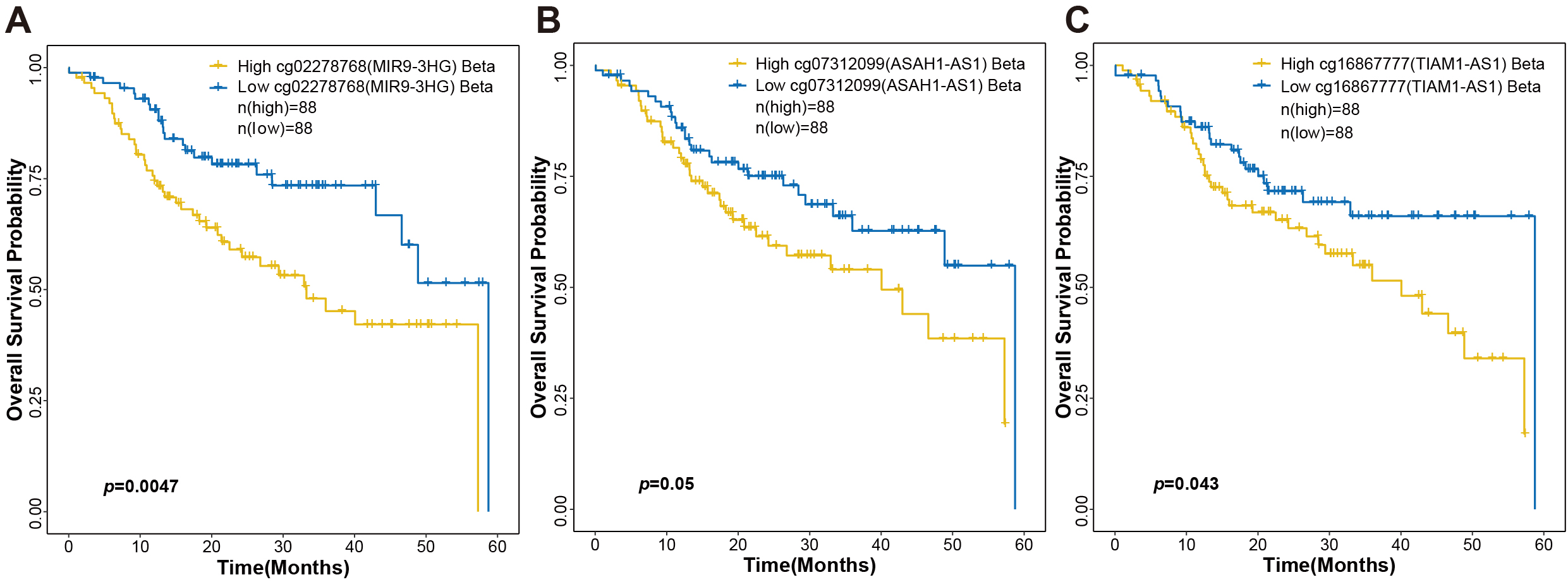

Supplement: Supplementary Figure 3 — DNA methylation analysis validation of CRLs in HNSCC in validation cohort. (A–C) Kaplan−Meier OS curves for HNSCC patients based on cg02278768 (MIR9-3HG) (A), cg07312099 (ASAH1-AS1) (B) and cg16867777 (TIAM1-AS1) (C) methylation levels in validation cohort. [file Image_3.jpeg]

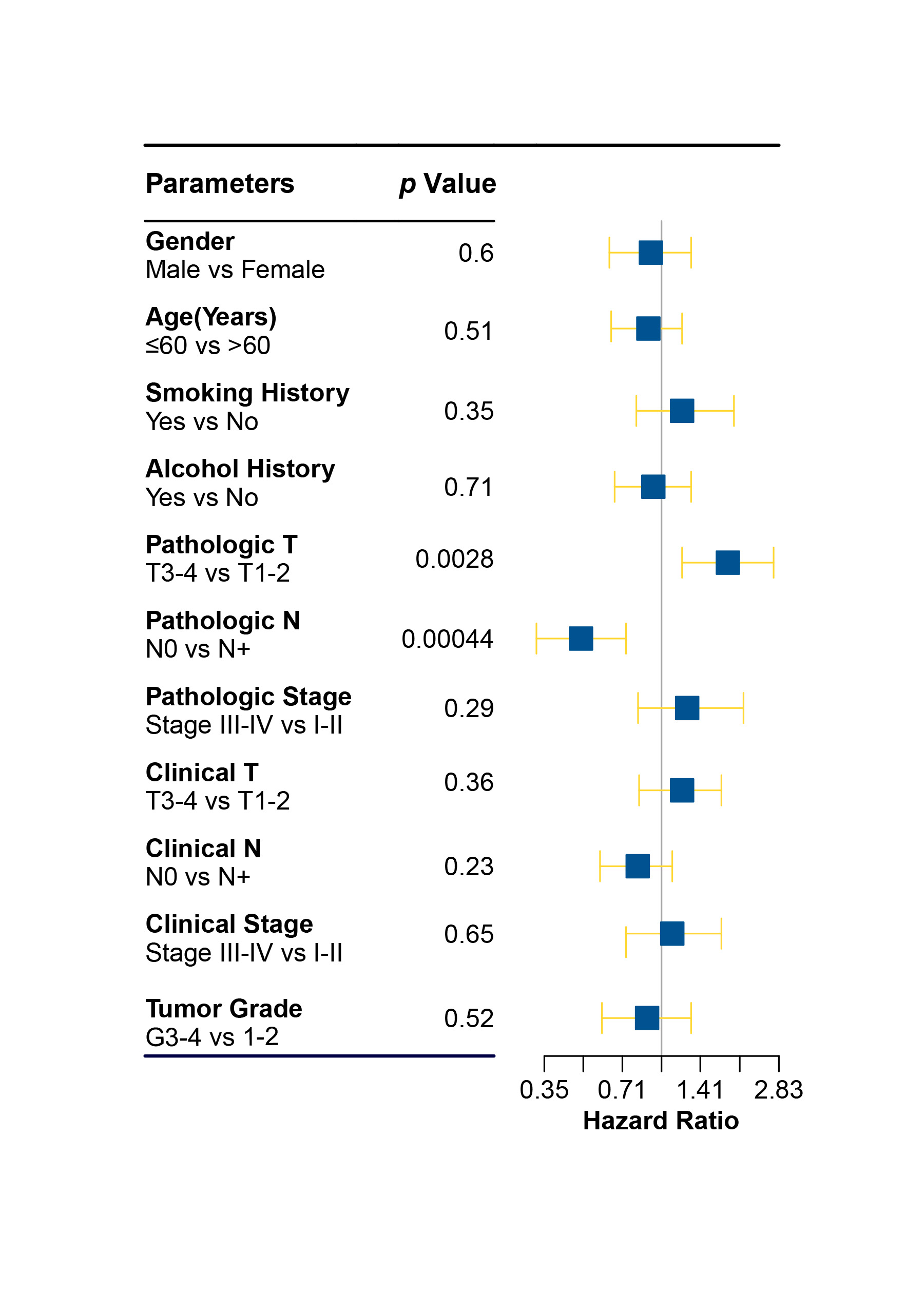

Supplement: Supplementary Figure 4 — Univariant Cox regression analysis of OS based on clinical features. [file Image_4.jpeg]
