# Supplementary material for: A cuproptosis-related lncRNA signature predicts the prognosis and immune cell status in head and neck squamous cell carcinoma
Source: Front Oncol. 2023 Jul 19;13:1055717. doi: 10.3389/fonc.2023.1055717 (PMC10394648; doi:10.3389/fonc.2023.1055717)
Supplement: Supplementary Table 1 — Clinical characters of HNSCC patients in the current study. [file Table_1.docx]

**Supplemental Table S1 Clinical characters of patients with HNSCC**

| **Parameters** | **Number of Cases** | |
| --- | --- | --- |
|  | **TCGA-A** | **TCGA-B** |
| **Gender** |  |  |
| Male | 213 | 154 |
| Female | 87 | 47 |
| **Age (Years)** |  |  |
| ≤60 | 146 | 99 |
| >60 | 154 | 101 |
| NA | 0 | 1 |
| **Clinical M** |  |  |
| M0 | 284 | 187 |
| M1 | 1 | 4 |
| MX | 13 | 7 |
| NA | 2 | 3 |
| **Clinical N** |  |  |
| N0 | 135 | 104 |
| N1 | 49 | 31 |
| N2 | 100 | 53 |
| N3 | 3 | 4 |
| NX | 11 | 7 |
| NA | 2 | 2 |
| **Clinical T** |  |  |
| T0 | 0 | 0 |
| T1 | 20 | 13 |
| T2 | 79 | 65 |
| T3 | 80 | 50 |
| T4 | 112 | 67 |
| TX | 7 | 4 |
| NA | 2 | 2 |
| Clinical Stage |  |  |
| Stage I | 11 | 8 |
| Stage II | 51 | 44 |
| Stage III | 61 | 41 |
| Stage IV | 169 | 102 |
| NA | 8 | 6 |
| **Pathologic T** |  |  |
| T0 | 0 | 1 |
| T1 | 20 | 25 |
| T2 | 81 | 52 |
| T3 | 61 | 35 |
| T4 | 107 | 64 |
| TX | 31 | 24 |
| NA | 0 | 0 |
| **Pathologic N** |  |  |
| N0 | 100 | 71 |
| N1 | 37 | 28 |
| N2 | 107 | 58 |
| N3 | 3 | 4 |
| NX | 40 | 29 |
| NA | 13 | 11 |
| **Pathologic M** |  |  |
| M0 | 110 | 77 |
| M1 | 0 | 1 |
| MX | 36 | 25 |
| NA | 154 | 98 |
| **Pathologic Stage** |  |  |
| Stage I | 11 | 14 |
| Stage II | 43 | 30 |
| Stage III | 46 | 32 |
| Stage IV | 167 | 93 |
| NA | 33 | 32 |
| **Smoking History** |  |  |
| Yes | 219 | 161 |
| No | 73 | 38 |
| NA | 8 | 2 |
| **Alcohol History** |  |  |
| Yes | 197 | 136 |
| No | 95 | 62 |
| NA | 8 | 3 |
| **Tumor Grade** |  |  |
| G1 | 30 | 31 |
| G2 | 190 | 110 |
| G3 | 68 | 51 |
| G4 | 0 | 2 |
| GX | 9 | 7 |
| NA | 3 | 0 |
